# Bioprinting Methods for Fabricating In Vitro Tubular Blood Vessel Models

**DOI:** 10.34133/cbsystems.0043

**Published:** 2023-08-01

**Authors:** Seon-Jin Kim, Min-Gyun Kim, Jangho Kim, Jessie S. Jeon, Jinsoo Park, Hee-Gyeong Yi

**Affiliations:** ^1^ Department of Rural and Biosystems Engineering, College of Agriculture and Life Sciences, Chonnam National University, Gwangju, Republic of Korea.; ^2^ Department of Convergence Biosystems Engineering, College of Agriculture and Life Sciences, Chonnam National University, Gwangju, Republic of Korea.; ^3^ Interdisciplinary Program in IT-Bio Convergence System, Chonnam National University, Gwangju 61186, Republic of Korea.; ^4^ Department of Mechanical Engineering, Korea Advanced Institute of Science and Technology, Daejeon, Republic of Korea.; ^5^ Department of Mechanical Engineering, Chonnam National University, Republic of Korea.

## Abstract

Dysfunctional blood vessels are implicated in various diseases, including cardiovascular diseases, neurodegenerative diseases, and cancer. Several studies have attempted to prevent and treat vascular diseases and understand interactions between these diseases and blood vessels across different organs and tissues. Initial studies were conducted using 2-dimensional (2D) in vitro and animal models. However, these models have difficulties in mimicking the 3D microenvironment in human, simulating kinetics related to cell activities, and replicating human pathophysiology; in addition, 3D models involve remarkably high costs. Thus, in vitro bioengineered models (BMs) have recently gained attention. BMs created through biofabrication based on tissue engineering and regenerative medicine are breakthrough models that can overcome limitations of 2D and animal models. They can also simulate the natural microenvironment in a patient- and target-specific manner. In this review, we will introduce 3D bioprinting methods for fabricating bioengineered blood vessel models, which can serve as the basis for treating and preventing various vascular diseases. Additionally, we will describe possible advancements from tubular to vascular models. Last, we will discuss specific applications, limitations, and future perspectives of fabricated BMs.

## Introduction

Blood vessels are important components of the circulatory system. They are involved in various activities of organs in the human body. Oxygenated blood and nutrients are circulated by the heart through arteries to cells, tissues, and organs. These arteries branch into smaller vessels called arterioles as they move further away from the heart and end in capillaries, the smallest blood vessels, delivering oxygen and nutrients as well as collecting waste products. Blood containing waste products then circulates back to the heart through veins. Blood vessels are distributed throughout the body and play a key role in the absorption and supply of nutrients as well as the elimination of waste products. Abnormalities in this system can lead to pathological states such as severe dysfunctions and even death [1–3].

Cardiovascular diseases (CVDs) are the most common cause of death worldwide, accounting for 30% of total global mortality according to the World Health Organization, with an increasing annual trend [4]. CVDs arise when blood vessels are narrowed or obstructed, mostly due to reduced function of the vascular endothelium, and are specifically caused by accumulation of plaques in coronary arteries, narrowing vessels or causing formation of blood clots that block blood vessels. Similarly, neurodegenerative diseases are caused by dysfunctional blood vessels. Several attempts have been made to treat and/or prevent blood vessel-related diseases, including cardiovascular and neurodegenerative diseases [5–9]. In addition, blood vessels play a major role in cancer pathogenesis. The human body maintains homeostasis through angiogenesis. Nevertheless, when the balance is disrupted, diseases such as cancer may develop. Cancer cells can exploit blood vessels with structural defects due to abnormal angiogenesis to receive oxygen and necessary nutrients and metastasize throughout the body. Several studies have been conducted to prevent abnormal angiogenesis. Ongoing research studies are investigating the association between cancer of different organs and blood vessels [10–18].

Three-dimensional (3D) models in cell culture have become increasingly popular in recent years to study human physiology and diseases in a more relevant and accurate manner compared to 2D in vitro and animal models. Unlike 2D cell cultures, 3D cultures can better mimic the 3D microenvironment of human tissues and maintain the complex network of blood circulation, which is critical for cellular functions. Furthermore, 3D models can better reflect tissue-specific characteristics, biochemical and mechanical signals, and cell–cell interactions, which are often lost in 2D cell cultures [19]. On the other hand, animal models are limited in their ability to accurately replicate human pathophysiology due to their high cost, time consumption, differences among species, and ethical concerns. 3D models offer a more feasible alternative as they can closely mimic in vivo environment and regulate cell proliferation and differentiation in a physiological manner. Therefore, 3D models are becoming a more attractive option for studying human biology and diseases than traditional 2D cell cultures and animal models [20,21].

This has led to the development of in vitro bioengineered models (BMs) through biofabrication based on tissue engineering and regenerative medicine. BMs are innovative models that can overcome limitations of 2D and animal models to allow simulation of the natural microenvironment in the human body in a patient- and target-specific manner. For instance, organoids mimic real organs anatomically and can be used in organ development and disease treatment [22]. Organ-on-a-chip can recapitulate human physiology on a chip for drug discovery and screening [23]. Similarly, there are BMs on microfluidic chips. By adding an element of fluid flow to the environment where cells are cultured in 3 dimensions, it is possible to coculture various cells while controlling spatial and temporal gradients of soluble factors for delicate manipulation. A high-resolution patterned culture substrate can be precisely designed in a desired shape. The flow of fluid with various characteristics can be simulated by controlling the diameter of the channel, the speed of the fluid, and so on. The microfluidic chip, which can continuously supply nutrients to the chamber, enables smooth removal of by-products generated by cell metabolism. It is useful for bioactivity analysis and toxicity evaluation of drugs that require repeatability [24–28]. These models are advantageous in that they can mimic in vitro structures, functions, and microenvironments of cells, tissues, and organs in the human body [29]. Specifically, BMs can be used to verify the safety and efficacy of drugs and medical devices by recreating structures and functions that maximally resemble those of tissues and organs in vitro. Furthermore, a new paradigm is proposed, whereby artificial organs can be produced by BMs in the organ transplantation market, with the demand far exceeding the supply [30–40].

One biofabrication method is 3D bioprinting. This 3D bioprinting technology is important because it allows creation of 3D forms of biologically relevant structures that mimic the complexity of in vivo environments more closely than 2D and animal models. Unlike traditional 2D cell cultures, 3D bioprinted structures can better represent the microarchitecture and microenvironment of human tissues and organs, enabling the study of cellular behavior, tissue development, and disease progression in a more realistic way. Additionally, 3D bioprinting provides a platform for personalized medicine as bioprinted tissues can be customized to match a patient's unique anatomy, allowing for more precise and effective treatments. Moreover, 3D bioprinting can revolutionize drug discovery and development process as it allows drug testing on functional human tissues before moving to clinical trials, reducing the need for animal testing and increasing the likelihood of success in human trials. Overall, 3D bioprinting technology provides a more precise and effective means for investigating biological processes and developing new treatments than traditional 2D cell cultures. Therefore, it is a crucial tool in the field of regenerative medicine and biological research.

In this review, we will introduce 3D bioprinting methods for fabricating bioengineered blood vessel models that underlie the treatment and prevention of blood vessel-related diseases and describe potential progression from tubular to vascular models. Additionally, we will discuss specific applications, limitations, and future perspectives for fabricated bioengineered blood vessel models.

## Pioneering Efforts for Fabricating Tissue-engineered In Vitro Blood Vessel Models

### Hydrogel casting method

Hydrogel casting is a method used in tissue engineering and regenerative medicine to create tubular structures such as blood vessels, trachea, and urethras. The process involves mixing a hydrogel such as gelatin or alginate (Alg) hydrogel with cells and then casting the mixture into a mold in the shape of a tube. The mold can be made of a variety of materials, including silicone, polycarbonate (PC), and other biocompatible materials. These materials can be easily removed after the casting process. The hydrogel provides a 3D environment for cells to grow and form a functional tissue, while the mold restricts the shape of the tissue and allows formation of a tubular structure. These cells within the hydrogel can form an extracellular matrix, which provides mechanical support and helps maintain the shape of the tube. Hydrogel casting is a relatively simple and cost-effective method for creating tubular structures. It is widely used in research and development for tissue engineering applications. This method can be modified to incorporate different materials and cells. It can be used to create functional biological structures with varying mechanical properties, biological properties, and functionalities.

**Fig. 1.**
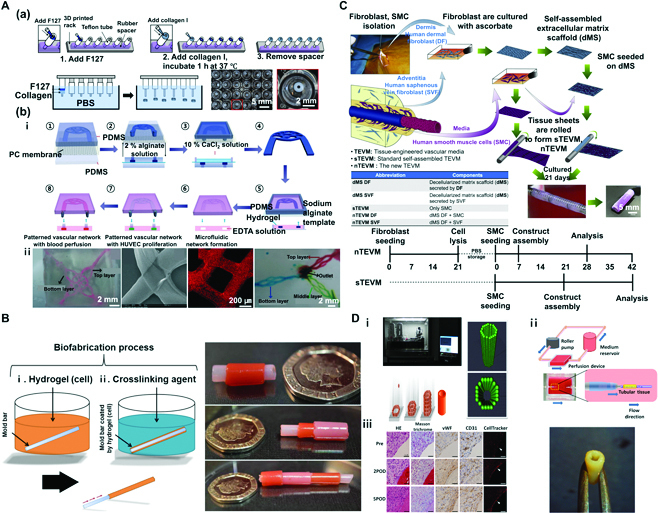
(A) Hydrogel casting method. (a) Method 1: Fabrication of freestanding cell-laden hydrogel blood vessel rings using a casting mold. Reproduced with permission [41]. Copyright 2022, The Authors. (b) Method 2: Fabrication of interconnected 3D vascular network construct using a sacrificial template and a casting mold. (i) Schematic diagram of the fabrication process. (ii) An image of a 3D vascular network construct in gelatin hydrogel, an image of a perfusion test using red microspheres, and an image of 3-layer interconnected vascular networks. Reproduced with permission [42]. Copyright 2014, The Royal Society of Chemistry. (B) Micro-dip coating method. Schematic diagram of tubular structure biofabrication process via dip coating. Reproduced with permission [43]. Copyright 2017, The Authors. (C) Extracellular sheet matrix rolling assembly method. Schematic diagram of biofabrication process of a tubular structure via sheet rolling, and timeline required to produce vascular constructs for comparing nTEVM (the new TEVM) versus sTEVM (standard self-assembled TEVM). Reproduced with permission [44]. Copyright 2012, Elsevier. (D) Tubular structures fabricated using the Kenzan method. (i) The system of lamination of MCS (Bio-3D printer) involves skewering of MCSs into needle array according to a 3D structure predesigned using a computer system. (ii) Schematic illustration of a bioreactor system and scaffold-free vascular graft generated from MCSs. (iii) Graft post-implantation is patented and remodeled. Reproduced with permission [45]. Copyright 2015, The Authors.

In the fabrication process demonstrated by Dostie et al. [41], a stereolithography (SLA)-based 3D printer was used to prepare a device rack with a rubber spacer fitted to the bottom. A commercial Teflon tube (outer diameter of 6.35 mm and inner diameter of 3.175 mm) was then placed on the spacer as a mold, which could prevent changes in hydrogel shapes. It could be easily removed. For adequate shaping through sealing of the space between the rack and the Teflon tube to prevent the release of collagen, Pluronic F127 (PF 127) was applied for 5 min for polymerization at 37 °C [Fig. 1A(a)] [41]. Next, human umbilical vein endothelial cells (HUVECs) or human umbilical artery smooth muscle cells (HUASMCs) were used to encapsulate collagen. Cell-containing collagen hydrogel was then pipetted on the polymerized PF 127. When PF 127 was adequately hard to support collagen, the spacer was removed, and the entire device was incubated at 37 °C for an hour to allow complete hardening of cell-containing hydrogel. Last, to obtain a hollow, ring-shaped hydrogel, the device was immersed in 1× phosphate-buffered saline (PBS), and the Teflon tube was pushed upward to expose PF 127 and collagen hydrogel to 1× PBS. This allowed removal of the exposed sacrificial PF 127 and formation of polymerized ring-shaped collagen. Outer and inner diameters of the obtained ring hydrogel were 2.6 to 4.6 mm and 0.79 to 1.0 mm, respectively. Both single-layer hydrogel containing HUVECs and hydrogel containing HUASMCs exhibited outstanding cell viability. In the fabrication process outlined by Dostie et al., the core was formed using an SLA 3D printer as performed in the first method. It was then immersed in 1 wt% bovine serum albumin (BSA) for 40 min to ensure easy removal of collagen without adhesion. The resulting 3D-printed core was fitted to a syringe. HUASMCs were used to encapsulate collagen. Subsequently, cell-containing hydrogel was carefully pipetted onto the core without any foam. Next, after approximately 1.5 h of incubation at 37 °C, the core was pushed upward to obtain a ring-shaped collagen vasculature. This method allows simple and rapid fabrication of vascular structures with free shapes.

In the second fabrication process shown by Wang et al. [42], a sodium alginate (Na-Alg) sacrificial mold was formed by combining a polydimethylsiloxane (PDMS) layer with a patterned structure for Na-Alg, a PDMS layer to which CaCl_2_ solution was applied, and a porous structure of PC with a pore size of 0.2 μm. Specifically, 2.0% (w/w) Na-Alg solution was placed on the top PDMS layer after structure patterning. With diffusion of 10% (w/w) CaCl_2_ solution to the bottom PDMS layer via the PC membrane, Alg polymerization occurred, leading to the production of a Na-Alg sacrificial template [Fig. 1A(b)-i: ① to ④]. Next, the Alg template was placed in a pre-gelled matrix hydrogel (gelatin, agarose, and collagen) for gelation. Reversible degradation of Alg occurred via strong chelation between Ca^2+^ and ethylene diamine tetra-acetic acid (EDTA). Removal of the Alg template in the hydrogel by addition of EDTA led to the fabrication of vascular structures with a microfluidic network [Fig. 1A(b)-i: ⑤ to ⑧] [42]. After encapsulation of the pre-gelled matrix hydrogel with high-density HUVECs, fully developed endothelial layers can be formed via perfusion, whereas HUVECs may adhere to the set microchannel. Analysis of the viability of HUVECs in fabricated vascular structures by calcein AM staining showed ≥99% viability after 24 h. Under static conditions, compared to those observed with flow conditions used with shear stress, cells in the fabricated vascular structures were shown to align parallel to the direction of flow. In addition, by assembling a Na-Alg template in different directions, interconnected 3D vascular networks were produced via multilayer fabrication to mimic in vivo cell growth. It is possible to construct perfusable, multilayer 3D vascular models by depositing 2 different Na-Alg templates, dissolving the resulting structure in EDTA solution, and creating interconnected junctions via Ca^2+^-mediated re-crosslinking [Fig. 1A(b)-ii]. Using the same method in which 3 different Na-Alg templates were deposited in different directions, perfusable triple-layer 3D vascular models were constructed. The team claimed that via the proposed hydrogel casting method, vascular structures of an outstanding level of cytocompatibility could be fabricated using Alg with high biocompatibility as a sacrificial template. Furthermore, the method is important in developing microenvironments that resemble native blood vessels in multilayer vascular structures, which could be used in a wide array of projects in tissue engineering and drug screening.

### Micro-dip coating method

Dip coating is a simple and widely used technique for coating surfaces with a thin film of a material, such as a polymer or a biological material. The process involves immersing a substrate into a liquid solution containing the material to be coated. The substrate is then slowly withdrawn from the solution, allowing a thin film of the material to be deposited on the surface. This method is widely used in fields of materials science, biotechnology, and tissue engineering. It is particularly useful for creating uniform, thin films over large areas.

Tabriz et al. [43] placed a tube-shaped stainless steel mold bar in cell-containing hydrogel for thin coating, transferred the bar to a solution of crosslinking agent to define the tubular shape, and removed the bar to fabricate tubular structures. In this method, Alg was used as the main substrate. As previously described, Alg is highly biocompatible and easy to use. Hence, it is widely used in biofabrication for tissue engineering and regenerative medicine. Materials used in a cell-laden hydrogel were 6% (w/v) Na-Alg solution and 0.4% (w/v) collagen solution. Mouse dermal embryonic fibroblasts were also used. Crosslinking agents used were 55 mM BaCl_2_ and 100 mM CaCl_2_. The detailed method of fabrication is described as follows. A sterilized stainless metal mold bar was dipped for 3 s in 6% (w/v) Na-Alg solution or hydrogel containing a mixture of 0.4% (w/v) collagen solution and 6% (w/v) Na-Alg solution. The bar was then dipped for 2 min in a solution containing crosslinking agent (55 mM BaCl_2_ or 100 mM CaCl_2_). Next, the molding bar was carefully pulled out to fabricate a hollow tubular hydrogel structure (Fig. 1B). After encapsulation with mouse dermal embryonic fibroblasts in Na-Alg solution, a tubular cell-laden hydrogel structure was fabricated. As with various previously mentioned hydrogel types, cells could be included in the fabrication process. Single-layer tubular Alg hydrogel structures of varying diameters can be fabricated using a previously described method. These single-layer tubular structures were formed by placing stainless steel rods in a cellular Alg solution. Tubular structures with diameters of 0.6, 1.2, 2.5, 3, 4, and 6 mm were obtained. Next, a perfusion test was performed on the Alg tubular hydrogel structure formed using a 4-mm rod. The flow rate of the red dye in the syringe pump was set at 20 ml/s for 3 min. To check leakage, a perfusion test was run with the structure placed above water. The result confirmed successful perfusion without any leakage. Using the previously described method, multilayer tubular structures can be fabricated, in addition to structures of varying diameters. To define conditions for cell survival and high proliferation rates, an experiment was conducted using hydrogel and crosslinking agents under varying conditions with mouse embryonic dermal fibroblasts (DFs). Tubular structures to be tested were fabricated via the previously described protocol using a 1.2-mm rod. The resulting tubular cell-laden hydrogel structure was then cultured for 6 days. These cell-laden hydrogels used in this experiment were Alg and collagen/Alg hydrogels. Crosslinking agents were BaCl_2_ and CaCl_2_ (Alg + barium; collagen/Alg + barium; Alg + calcium; collagen/Alg + barium). Analysis of cell viability using the live-dead assay showed that the cell viability of cultured tubular cell-laden hydrogel structures was high under all hydrogel and crosslinking conditions. However, slightly low cell viability was observed for structures polymerized using collagen/Alg hydrogel and BaCl_2_ and CaCl_2_ agents. Under the aforementioned 4 conditions, cells could grow and differentiate on fabricated tube walls. Here, the optimal condition of hydrogels and crosslinking agents should allow permeability for an adequate supply of O_2_ and nutrients with waste discharge. Results showed that cells formed an adequate extracellular matrix (ECM) via cell-to-cell interactions in the gel. Cell density results indicated that the cell-laden Alg solution obtained via polymerization using BaCl_2_ showed the highest cell density. Compared to other complicated and expensive tubular fabrication methods or machine-based systems, the novel and rapid 3D biofabrication technique is anticipated to simulate a wide range of human tubular tissues (embryonic kidney, lymph vessels, blood vessels, trachea, and intestine).

### Sheet matrix rolling assembly method

Cell sheet rolling is a technique used in regenerative medicine and tissue engineering to create functional biological structures. It has been used to create functional blood vessels, tracheal tubes, and other types of tubular structure. The process can be performed in a simple and efficient manner. It has the potential to be scaled up for use in large-scale tissue engineering applications.

Bourget et al. [44] constructed cell-laden matrix tubular vascular structures by fabricating an ECM-based, sheet-line matrix scaffold with seeded cells via rolling. The method of sheet fabrication involved isolation of saphenous vein fibroblasts (SVFs) from DFs of the dermis of a patient in the adventitia with subsequent mixing of Dulbecco-Vogt-modified Eagle’s medium (DMEM) and Ham’s F12 at a 3:1 ratio to form DMEM-Ham. This was mixed with 10% fetal calf serum (FCS), antibiotics, and 50 μg/ml of sodium ascorbate for culturing. On day 21 of culture, washing with deionized sterile water revealed the formation of decellularized matrix scaffold (dMS) DF or SVF. Smooth muscle cells (SMCs) of vascular media were seeded on the obtained dMS DF or dMS SVF. Similarly, sheets were assembled via rolling, whereby new TEVM (tissue-engineered vascular media) (nTEVM) was produced. It could be distinguished from standard self-assembled TEVM (sTEVM) obtained through rolling of sheets after seeding only SMCs on dMS (Fig. 1C). The dMS SMCs require 14 to 21 days for seeding and formation of self-assembled sheets. However, seeding of SMCs on dMS DF or dMS SVF allows assembly of the construct within 1 to 2 weeks to reduce total fabrication time while still allowing analyses. In addition, compared to constructs fabricated using other methods (sTEVM, dMS DF, and dMS SVF), novel engineered tissues (nTEVM DF and nTEVM SVF) fabricated using a dermal or vascular source demonstrated potent physical properties of ultimate tensile strength (UTS), modulus, burst pressure (BP), and failure strain. In Masson’s trichrome staining of the fabricated vascular constructs, the formation of a decellularized matrix (ECM sheet) for the dMS DF or dMS SVF was confirmed (dense collagen structure: blue), and the complete cohesion of SMCs on the dense ECM (dMS) could be observed with nTEVM DF, nTEVM SVF, and sTEVM. In immunofluorescence staining and analyses using SMC markers [α-smooth muscle actin (SMA) and calponin], a contractile SMC phenotype was observed for nTEVM DF, nTEVM SVF, and sTEVM. In this study, coronary artery bypass graft (CABG) was used to treat CVDs despite the fact that the gold standard in most protocols was implantation of internal mammary artery (IMA) or patient-derived saphenous vein (SV) and synthetic materials (for example, Dacron and expanded polytetrafluorethylene). However, due to drawbacks related to availability, time consumption, and immune rejection, patient-derived implantable cells were used to form a dMS (tunica externa), and SMCs (tunica media) were cultured to fabricate nTEVM of double layers (tunica externa and tunica media). While the method is highly interesting, it should be validated for the fabrication of vascular structures in the future.

### Kenzan method

The Kenzan printing method involves printing of spheroids on a microneedle array surrounded by a scaffold-free concentric structure for deposition, followed by fabrication of a single 3D structure via self-assembly of spheroids. Itoh et al. [45] applied the Kenzan method for the development of multicellular spheroids (MCSs) to create tubular tissues (Fig. 1D). MCSs are concentric spheroids that combine HUVECs, human aortic smooth muscle cells (HASMCs), and human normal dermal fibroblasts (HNDFBs) at a 4:1:5 ratio. By adequately depositing MCSs on fine needles (outer diameter: 0.17 mm; distance between needles: 0.4 mm), MCSs were aligned in a tube shape using a robotically controlled fine suction nozzle. The needle array was removed after 4 days of culture to reveal a single, converged tubular structure with a diameter of 1.5 mm (Fig. 1D-i). In a perfusion test, the resulting tubular structure was fitted to a bioreactor, and perfusion was confirmed (Fig. 1D-ii). An in vivo assay using nude rat aorta confirmed the production of endothelium on the inner luminal surface after implantation (Fig. 1D-iii). The fabrication of tubular structures using the Kenzan method was associated with challenges in the production of complex structures such as branched or bent blood vessels. The mechanical strength also deviated from that of native blood vessels. However, advantages of this method include a short time required for designing the size and shape of implantable tubular structures and facilitation of tissue fabrication under conditions that minimize contamination.

Although these fabrication methods for tubular structures are fascinating, they have several drawbacks. Many of these methods used to create functional tubular structures such as scaffold-based methods, cell sheet engineering, and hydrogel casting can be time-consuming and labor-intensive, making it difficult to scale up to meet demands of large-scale tissue engineering applications. In addition, materials used in these methods, such as synthetic scaffolds and hydrogels, can have limited mechanical and biological properties, making it challenging to create functional tubular structures with desired native tissues’ properties and functions. Furthermore, creating functional tubular structures often requires a complex combination of materials, cells, and methods, posing a challenge to control the quality and consistency of resulting structures. In addition, there is still a significant gap between the development of functional tubular structures in the laboratory and their translation into clinical applications. Thus, more work is needed to overcome these challenges associated with clinical translation. Despite these drawbacks, researchers continue to make progress in advancing these methods to create functional tubular structures using 3D bioprinting, a rapidly evolving field in tissue engineering and regenerative medicine. 3D bioprinting involves the use of advanced printing technologies to create functional biological structures such as tissues and organs using a combination of biological materials and cells. One of the key benefits of 3D bioprinting is its ability to control spatial arrangement and organization of cells and materials at micro- and nanoscale. This enables researchers to create highly complex and functional structures that are not possible to create using traditional tissue engineering methods. Additionally, 3D bioprinting allows precise and consistent production of structures, making it a promising technology for developing new therapies and treatments. There are several different approaches to 3D bioprinting, including extrusion-based bioprinting, ink jet bioprinting, support bath-based bioprinting, and laser-assisted bioprinting.

## 3D Bioprinting Techniques for Fabricating Tubular Structure to Create Tissue-engineered In Vitro Blood Vessel Models

### Microextrusion bioprinting

**Fig. 2.**
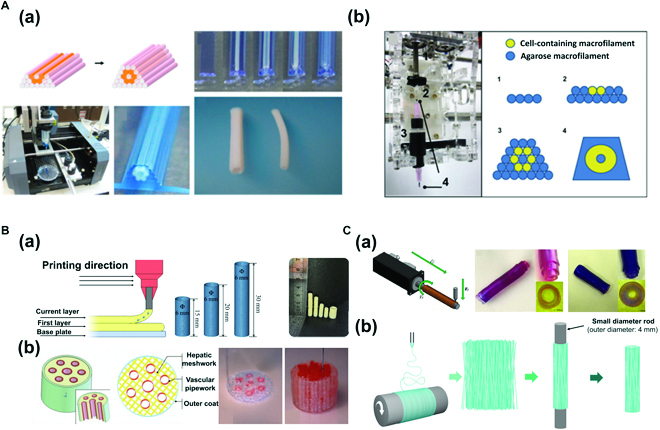
(A) Microextrusion bioprinting: (a) Fabrication of scaffold-free vascular tissue using agarose rods and cellular cylinders. Reproduced with permission [46]. Copyright 2023, Elsevier. (b) A Fab@Home printing system and printing protocol for building a cellular tubular structure by depositing hydrogel macrofilaments in additive manufacturing. Reproduced with permission [47]. Copyright 2010, Elsevier. (B) Concentric ring bioprinting. (a) Method 1: Direct concentric ring 3D printing using gelatin (Gt)-Alg-montmorillonite (MMT) bioinks. Images of the concentric ring printing method, 3D model, and 3D printed Gt-Alg-MMT hydrogel vascular scaffold. Reproduced with permission [48]. Copyright 2022, Elsevier. (b) Method 2: Modeling of liver tissue construct with a vascular network using direct concentric 3D printing method with gelatin/Alg/chitosan (GAC) hydrogel. Reproduced with permission [49]. Copyright 2009, SAGE Publications. (C) Rod bioprinting. (a) Formation of vascular structure and multimaterial vascular structure. Schematic image of a rod printer and rotating rods of various diameters. The relationship between thickness of the vascular structure and rod diameter is also shown. Printing hydrogel, multilayer vessel structure. Reproduced with permission [50]. Copyright 2021, The Authors. (b) Fabrication of artificial poly (lactic acid) (PLA) blood vessels using electrospinning and 3D rod printing. Reproduced with permission [51]. Copyright 2021, The Authors, licensed under a Creative Commons Attribution 3.0 International License.

In tissue engineering, scaffolds could induce unfavorable host responses, interfere with cell-to-cell interactions, and prevent the assembly and alignment of cell-produced ECM. Hence, Norotte et al. [46] printed agarose rods using rapid prototyping bioprinting and used these rods to fabricate “scaffold-free” vascular tissues (Fig. 2A). They used agarose rods as the molding template to build single and double layers for fabricating scaffold-free vascular tissues and further fabricated branched vascular tissues [Fig. 2A(a)]. MCSs [human umbilical vein smooth muscle cells (HUVSMCs) and human skin fibroblasts (HSFs)] were seeded on agarose rods. With gradual fusion of spheroids, tubular structures were formed within 5 to 7 days. Each of the 2 printer heads was used to print multicellular cylinders and agarose rods, respectively. Removal of agarose rods after fusion of spheroids (2 to 4 days) left only tubular structures. The same method was applied to fabricate double-layer blood vessels consisting of HUVSMC and HSF cylinders. However, in the study by Norotte et al. [46], fabricating microsized vascular structures remained a challenge. The diameter of the smallest blood vessel was 900 μm in size. Vascular structures of complex shapes could not be fabricated. Nevertheless, the significance lies in applying an automated process to fabricate a scaffold-free tubular structure and using spheroids to fabricate a tissue with a high level of biological features.

Similarly, Skardal et al. [47] developed tetra-acrylate derivative (TetraPAc)-based hydrogels to produce grafts that mimic native blood vessels and generated macrofilaments to fabricate tubular structures [Fig. 2A(b)]. These hydrogels developed through TetraPAc crosslinking demonstrated far higher elasticity and hardness than polyethylene glycol diacrylate (PEGDA) crosslinking. TetraPAc-based hydrogels were suitable for printing high-density cells. Equivalent or more outstanding levels of cell growth and proliferation compared to those with PEGDA were observed. These hydrogels developed using TetraPAc8 and TetraPAc13 were dissolved in collagen/hyaluronidase to obtain TectraPAc13-crosslinked hydrogels, which were encapsulated with NIH 3T3 cells (25 × 10^6^ cells/ml). These hydrogels were placed in macrocapillary tubes with a diameter of 5 μm for crosslinking. In addition, agarose was heated and dissolved in PBS to generate agarose macrofilaments. Each macrofilament was loaded to the printer for bioprinting. The structure was covered with a thin agarose layer immediately after printing to prevent separation of filaments. The resulting microfilament-based tubular structures containing cells exhibited substantially high cell viability after 4 weeks. In the study by Skardal et al. [47], a tubular structure with mechanical properties as outstanding as those of native blood vessels and a high biocompatibility was fabricated using macrofilaments.

#### Concentric ring bioprinting

Concentric ring bioprinting is a method used to fabricate tubular structures through stacking of concentric rings, in which the core principle of 3D bioprinting to fabricate 3D structures via layer-by-layer bioink stacking is applied. Wu et al. [48] have fabricated hollow tubular structures through repeated stacking of ring-shaped bioink. By controlling printing parameters (layer thickness, height of syringe head, and speed of syringe head), the size and radius of the target tubular structure can be adjusted [Fig. 2B(a)] [48]. The advantage of using this method is that it can fabricate 3D structures in a short period of time. In the study of Wu et al. [48], bioink combining Gt-Alg-MMT was used. Cell viability was excellent. Fabricated vascular structures exhibited outstanding mechanical strength (tensile strength and BP) compared to other synthetic polymers, natural synthetic polymers, or native arteries. In addition, Li et al. [49] have produced an artificial liver using the concentric 3D printing method to fabricate cell-laden hydrogel-based anatomical liver structures with a vascular network to supply adequate quantities of oxygen and nutrients [Fig. 2B(b)]. The concentric ring printing technique allows for precise control of cell placement, distribution, and organization within the printed structure. This in turn enables creation of tissues and organs with realistic microarchitecture and improved mechanical and biological functionality. Concentric ring 3D bioprinting has a wide range of potential applications, including development of functional tissues for drug screening and toxicity testing and creation of replacement tissues and organs for transplantation. Overall, concentric ring 3D bioprinting is a promising and innovative approach for creation of functional biological structures. It holds great potential for advancing the development of new therapies and treatments for a variety of medical conditions.

#### Rod bioprinting

Rod bioprinting involves printing of cell-containing bioink on rotating rods. Liu et al. [50] used this method to fabricate hollow structures by first depositing uniform patterns on rotating rods as the printer moved along *s* and *y* axes with rods subsequently removed. This method allows control of rod rotation speed and magnitude, extrusion intensity and speed, and other parameters for fabricating tubular structures of varying shapes and diameters. Vascular structures can be fabricated by printing hydrogels containing different cells to mimic the characteristic multilayer structure of blood vessels on the rods rotating in a step-by-step manner [Fig. 2C(a)] [50]. An electrospinning 3D rod printing method in which polymers can be patterned rapidly by applying electrospinning to polymers released from microextrusion has also been reported. A high voltage is applied to the extruded polymer solution from the syringe for coating of nanofiber polymers. Nanofiber tubular structures of micropolymers can be fabricated with the removal of rods [Fig. 2C(b)] [51]. The rod-shaped printing head allows more precise and reproducible deposition of cells and biomaterials, which is important for creating functional tissue structures with a well-defined architecture. This technology has been used in research to create functional in vitro models of various tissues as well as vascular tissue, including skin, bone, and muscle. It has the potential applications as regenerative medicines in the future.

#### Coaxial nozzle bioprinting

Coaxial nozzle microextrusion is a 3D bioprinting technique used to fabricate structures of concentric shapes via simultaneous printing of bioink of 2 different materials. In general, sacrificial bioink is applied to the concentric core for printing. Its removal generates hollow tubular structures. By adjusting printing parameters (such as printing speed and air pressure) and nozzle diameter, tubular structures of varying sizes and shapes can be fabricated with not only temporal and economic efficiency but also high level of reproducibility and repeatability. This technique also allows precision printing to mimic characteristics of native blood vessels via mechanical control.

**Fig. 3.**
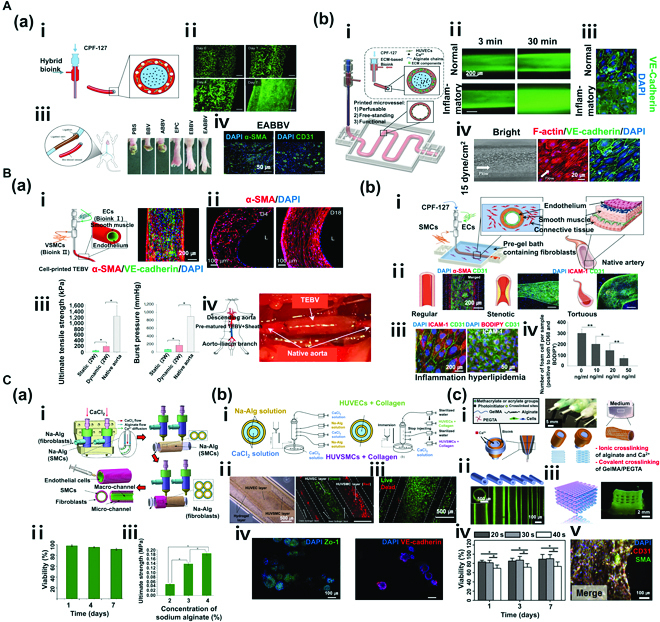
(A) Coaxial nozzle bioprinting (monolayer). (a) Tissue-engineered bio-blood vessels developed using coaxial nozzle cell printing for ischemic disease. (i) Schematic diagram of coaxial nozzle and materials. (ii) Live/dead assay for printed EPC (endothelial progenitor cell)-laden BBV during 7 days. Cells were stained with calcein AM (live, green) and ethidium homodimer I (dead, red). (iii) Representative images of 6 groups (PBS, BBV, ABBV, EPC, EBBV, and EABBV). (iv) Immunostaining results of EPC/APMS-laden BBV. The 6 groups were stained with anti-CD31 antibody and anti-α-SMA antibody after 28 days. BBV, bio-blood vessel; ABBV, atorvastatin-loaded poly (lactic-co-glycolic) acid microsphere (APMS)-laden BBV; EBBV, EPC-laden BBV; EABBV, EPC/APMS-laden BBV. Reproduced with permission [52]. Copyright 2017, The Authors, licensed under a Creative Commons Attribution 4.0 International License. (b) Freestanding, perfusable, and functional in vitro vascular models developed using coaxial nozzle cell printing for mimicking native endothelium pathophysiology. (i) Schematic diagram of coaxial nozzle cell printing process. (ii) Permeability test for a normal vascular model (VM) and an inflammatory model. (iii) Immunostaining results of normal and inflammatory VM indicating disruptions of the endothelial barrier in the inflammatory model. (iv) Response to shear stress in VM. Reproduced with permission [53]. Copyright 2018, WILEY-VCH Verlag GmbH & Co. KGaA, Weinheim. (B) Coaxial nozzle bioprinting (multilayer). (a) Tissue-engineered vascular grafts containing an endothelial layer and a muscle layer using triple-coaxial cell printing technology. (i) Schematic diagram of triple-coaxial nozzle, materials, and tissue-engineered blood vessels (TEBVs), and immunostaining results of TEBV. (ii) Culturing of TEBV under static conditions (left) and dynamic conditions (right). (iii) Mechanical property test (UTS and BP). (iv) Abdominal aorta graft of TEBV in the rat model. Reproduced with permission [54]. Copyright 2019, AIP Publishing. (b) Fabrication of atherosclerotic in vitro model containing endothelium and smooth muscles. (i) Schematic diagram of the cell printing process, native artery, and materials. (ii) Triple-layer arterial constructs (regular, stenotic, and tortuous). (iii) Endothelial dysfunction response of the arterial construct [inflammation: tumor necrosis factor-α (TNF-α), hyperlipidemia: low-density lipoprotein (LDL)]. (iv) Evaluation of the effect of atorvastatin on an arterial construct (turbulent flow). Reproduced with permission [55]. Copyright 2020, Wiley-VCH GmbH. (C) Vessel-like structure with multilevel fluidic channels. (a) Fabrication process of Na-Alg cell-laden hydrogel vessel-like structure containing endothelium and smooth muscles using coaxial nozzle and rod printing methods. (i) Schematic of the fabrication process. (ii) Live/dead assay (live, green; dead, red) of vessel-like structure. (iii) Test of mechanical properties (UTS) of the construct. Reproduced with permission [56]. Copyright 2017, American Chemical Society. (b) Fabrication of multicellular vessel structure using 4 flow channels. (i) Schematic diagram of the 4-channel coaxial nozzle. (ii) Images showing the distribution of cells via cell-laden hydrogel perfusion. (iii) Live/dead assay (live, green; dead, red). (iv) Immunostaining image showing expression of ZO1 tight junction and VE-cadherin proteins in HUVECs and HUVSMCs. Reproduced with permission [57]. Copyright 2018, Elsevier. (c) Direct 3D bioprinting of perfusable vascular constructs. (i) Schematic diagram of various coaxial nozzle and materials. (ii) 3D-printed vascular constructs with various sizes. (iii) Perfusable 10 layers of a 3D bioprinted construct. (iv) Live/dead assay (live, green; dead, red) of vascular constructs. (v) Immunostaining images showing expression of CD31 and SMA. Reproduced with permission [58]. Copyright 2016, Elsevier.

Using this technique, Gao et al. [52] printed tissue-engineered blood vessel (TEBV) for the treatment of ischemic diseases by applying calcium chloride (CaCl_2_)/PF 127 solution (CPF 127) as a sacrificial bioink to the concentric core and combining hybrid bioink [a mixture of vascular tissue-derived decellularized extracellular matrix (VdECM) and Alg] with human endothelial progenitor cells, atorvastatin, and poly (lactic-co-glycolic) acid (PLGA) microsphere on the exterior [Fig. 3A(a)-i]. TEBV generated by combining human endothelial progenitor cells, atorvastatin, and PLGA microspheres displayed an outstanding viability of encapsulating cells with characteristics resembling native blood vessels [Fig. 3A(a)-ii, iv]. When this TEBV was implanted in ischemic limbs of mice, salvage of ischemic limbs was observed after 28 days [Fig. 3A(a)-iii]. In addition to blood vessels used to treat ischemic diseases, in vitro vascular models of free shapes and perfusability were produced to mimic the endothelium pathophysiology of native blood vessels [Fig. 3A(b)-i] [53]. These models were created by applying CPF 127 to the concentric core and the hybrid bioink (a mixture of VdECM and Alg) on the exterior. The resulting in vitro models were compared with in vitro inflammatory stimuli models with respect to immunofluorescence staining images and diffusional permeability. Normal in vitro models showed selective permeability in contrast to inflammation in in vitro models with disruptions of endothelial barriers [Fig. 3A(b)-ii, iii]. Physiological functions were also assessed by applying shear stress. Results showed an elongated morphology and alignment of cells [Fig. 3A(b)-iv].

Gao et al. [54] developed a 3D cell printing technology with triple-coaxial nozzles and constructed TEBV comprising endothelium encapsulated by HASMCs after several weeks of cell culture [Fig. 3B(a)-i, ii]. Blood vessels, particularly large arteries, are not simple hollow tubes but complex tissues that comprise vascular endothelial cells, connective tissues, and smooth muscle layers. Arteries, in particular, are unable to retain adequate pressure without muscles that contract according to blood pressure. When TEBV comprising muscle and endothelium layers was fabricated via 3D bioprinting and cultured under dynamic conditions (pulsatile stimulation) for 2 weeks, mechanical properties (UTS and BP) of these TEBVs were approximately 4-fold higher than those of TEBVs cultured under static conditions (no pulsatile perfusion) [Fig. 3B(a)-iii]. HASMCs were erratically aligned before culturing under dynamic conditions. However, after 2 weeks of culture under dynamic conditions, cells were circumferentially oriented along the curve of the vessel wall [Fig. 3B(a)-ii; left: before dynamic condition, right: after dynamic condition]. When cell-printed TEBV was grafted into the rat abdominal aorta to evaluate in vivo performance, the result was encouraging as TEBV could successfully recapitulate the function of the aorta [Fig. 3B(a)-iv]. The team has also developed an in-bath triple-axial printing technology for printing in a bioink bath containing fibroblasts in addition to implantation of multilayer blood vessels (endothelium and muscle layers) to produce functional arterial in vitro models (endothelium, muscle, and fibroblast layers) of varying geometric shapes loaded with a structurally stable fibroblast layer [Fig. 3B(b)-i]. These artificial arterial in vitro models showed changes in blood flow dynamics according to structural specificity. Turbulent blood flow created in stenotic and curved blood vessels led to dysfunction of endothelial cells [Fig. 3B(b)-ii]. When these models were treated with atorvastatin, a drug for vascular diseases, genes involved in activation of endothelial cells, white blood cell chemotaxis, macrophage activity, and cholesterol variation were expressed. These findings verified the utility of the model as a platform in drug analysis [Fig. 3B(b)-iii, iv] [55]. We speculate triple-layer artificial arterial in vitro model developed by Gao et al. [52] as a promising platform to elucidate the pathophysiology of atherosclerosis and discover effective treatments and drugs.

In addition to application of the sacrificial bioink CPF 127 to the concentric core of the coaxial nozzle and then removing it after printing, a method in which only CaCl_2_ solution is applied to create vascular structures has been developed [52]. For instance, combining Alg solution with divalent cations (e.g., Ca^2+^, Ba^2+^, and Mg^2+^) can result in rapid ionic crosslinking to produce a viscous hydrogel.

In the first method of biofabrication, CaCl_2_ solution is applied to the core and Na-Alg is applied to the shell for printing viscous Alg hydrogel on rotating rods to fabricate vascular structures. First, SMC is mixed with Na-Alg. Cell-laden Alg hydrogel is printed on rotating rods via simultaneous printing with CaCl_2_ solution. Next, on the same principle, fibroblasts encapsulated with Na-Alg via adjacent printing heads are printed on printed tubular structures. Subsequent immersion of multilayer vascular structures fabricated using the CaCl_2_ solution results in complete crosslinking. When multilayer vascular structures with robust mechanical properties are perfused by collagen-containing endothelial cells for a set period, complete vascular structures of the endothelium, muscle, and fibroblast layers can be fabricated [Fig. 3C(a)-i] [56]. These fabricated vascular structures exhibited excellent cell viability until day 7 [Fig. 3C(a)-ii]. By establishing a specific concentration of Na-Alg solution, the fabrication of vascular structures with outstanding mechanical strength was rendered possible [Fig. 3C(a)-iii].

The second method of biofabrication involves a 4-layer coaxial nozzle, where Na-Alg solution is applied to the second and fourth layers, and CaCl_2_ solution containing divalent cations for Na-Alg crosslinking is applied to the first and third layers. The printing is performed in a bath of CaCl_2_ solution. In the first printing process, printing starts within a range of approximately 50 mm from the surface of CaCl_2_ bath. Via induction of primary crosslinking by Na-Alg in the coaxial nozzle and secondary polymerization in CaCl_2_ bath, polymerization can be completed to create multilayer structures of structurally stable shapes. Next, channel flow to the 4 layers is blocked at the completion of 4-layer coaxial structures. Via instant immersion of the coaxial nozzle tip in CaCl_2_ bath, 4-layer coaxial structures are induced to adhere to the coaxial nozzle tip (Na-Alg solution + CaCl_2_ solution crosslinking). Via subsequent perfusion by cell-laden collagen (HUVECs and HUVSMCs), vascular structures comprising endothelium and muscle layers can be fabricated. Subsequently, the cells were found to be evenly distributed with viability of ≥90% [Fig. 3C(b)-i, ii] [57]. In addition, after 7 days of culture, steady growth and normal proliferation of cells were observed [Fig. 3C(b)-iii]. Monitoring cellular morphological changes also confirmed normal expression levels of ZO1 tight junctions and VE-cadherin proteins [Fig. 3C(b)-iv].

In the third method of biofabrication, direct printing of cell-laden hydrogel was used to fabricate perfusable vascular constructs, in contrast to perfusion by a cell-laden hydrogel in the first and second methods. The bioink in this method comprised blended hybrid bioink obtained by combining gelatin methacryloyl (GelMA), Na-Alg, and 4-arm poly(ethylene glycol)-tetra-acrylate (PEGTA). CaCl_2_ solution was applied for Na-Alg crosslinking to the first and third layers, and the hybrid bioink combining GelMA, Na-Alg, and PEGTA was applied to the second layer. Ionic crosslinking of NA-Alg by divalent cations (Ca^2+^) was induced, and photogenic crosslinking of GelMA and PEGTA was induced via light irradiation to fabricate structurally stable and perfusable vascular constructs [Fig. 3C(c)-i] [58]. Using coaxial nozzles of varying diameters, it was possible to form vascular structures of different inner and outer diameters [Fig. 3C(c)-ii]. Perfusion was confirmed after depositing lattice-shaped vascular structures for up to 10 layers [Fig. 3C(c)-iii]. In addition, by varying intervals of light irradiation, cell viability of fabricated vascular structures was compared; it was higher for the structure with a relatively shorter time of light irradiation than that of a structure subjected to light irradiation for the longest time of 40 s [Fig. 3C(c)-iv]. Analysis of CD31 and SMA biomarkers via immunofluorescence staining showed clearly visible biomarkers in these structures cultured for 21 days. Based on these results, the vascular structures fabricated with novel bioink and printing parameters were shown to be maturated vessels that effectively resembled the native vasculature with outstanding cell spreading and proliferation [Fig. 3C(c)-v].

Coaxial bioprinting allows precise control over placement and distribution of cells within the printed structure, which is important for creating functional tissues and organs with realistic microarchitecture. It minimizes mechanical stress on the cells during printing, which can help improve cell viability and reduce cell death. Coaxial bioprinting can also be used to print a wide range of cells and materials, including stem cells, primary cells, and hydrogels, making it a versatile method for creating functional biological structures. Furthermore, this technology can be scaled up to print larger structures such as whole organs, which is important for developing replacement tissues and organs for transplantation.

### Inkjet bioprinting

**Fig. 4.**
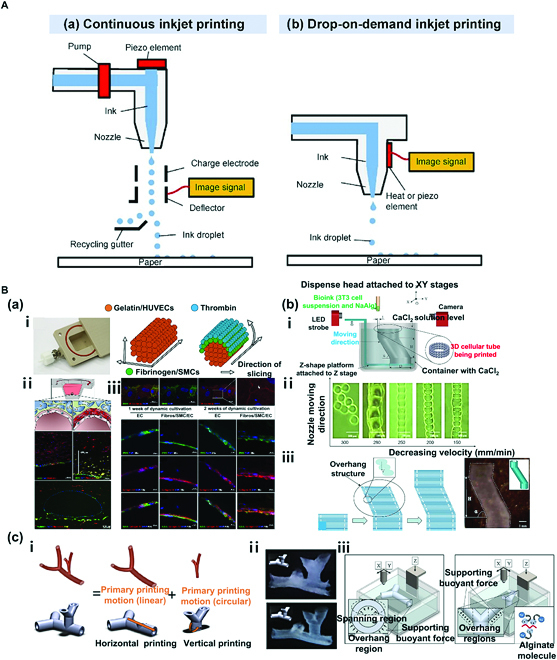
(A) Simplified representation of 2 different mechanisms of inkjet printing. (a) Continuous inkjet printing (CIJ) and (b) drop-on-demand printing (DOD). Reproduced with permission [60]. Copyright 2022, The Authors. (B) DOD inkjet bioprinting: (a) Fabrication of multilayered vessel using DOD inkjet bioprinting. (i) The cooling system of the bioprinter for optimal gelation timing (left) and detailed structure and shape of the printed tubular structure. (ii) Schematic diagram of the cross-section of the channel with a single layer of endothelial cells and fluorescence micrographs of vascular-like channels in the cross-section. (iii) Fluorescence micrographs of the endothelium in sections with qualitative and quantitative assessment of collagen IV expression [CD31: green; VE-cadherin and collagen IV: red; 4′,6-diamidino-2-phenylindole (DAPI): blue]. Reproduced with permission [62]. Copyright 2018, The Authors. (b) Fabrication of bifurcated vascular tree using DOD inkjet bioprinting. (i) Basic tubular structure of a vascular network. (ii) Horizontal and vertical bifurcations. (iii) CaCl_2_ solution provides a supporting buoyant force for spanning and overhang regions in horizontal printing and overhang regions in vertical printing. Schematic diagram of the gelation process is shown. Reproduced with permission [65]. Copyright 2012, Wiley Periodicals Inc. (c) Fabrication of zigzag-shaped overhang structure using DOD inkjet printing in CaCl_2_ bath. (i) Schematic of the proposed platform-assisted 3D inkjet bioprinting system. (ii) Gel lines printed using a 120-mm dispense head. (iii) Zig-zag tube printing process and a printed zigzag tube sample. Reproduced with permission [66]. Copyright 2014, Wiley Periodicals Inc.

Inkjet bioprinting is a technique that greatly reduces the loss of materials through extrusion of bioink based on the need. It entails relatively low cost and desirable printing resolution. As cells of varying types can be printed, this technique is one of the most widely used ones in the field of bioprinting. This technique also exhibits outstanding cell viability with lower stimulation of cells than that of other bioprinting techniques [59]. Inkjet printing is broadly divided into continuous inkjet printing (CIJ) and drop-on-demand (DOD) inkjet printing (Fig. 4) A [60]. In the CIJ method, bioink is extruded through an orifice of a small diameter with exerted pressure and consequent hydrodynamic instability, which results in creation of droplets [Fig. 4A(a)]. The bioink is printed until the process runs out of materials, while continuous pressure is applied to the fluid. As unused material is recycled via the gutter, which poses a risk of contamination, the CIJ is not a preferred method in bioprinting. On the other hand, DOD is a preferred method [61]. It involves application of pressure pulse based on thermal expansion or piezoelectric actuation to the syringe with bioink so that droplets can be created on demand and not continuously [Fig. 4A(b)]. The droplet diameter can be controlled to a microscale. Deposit of droplets leads to fabrication of 3D structures.

Nevertheless, DOD inkjet bioprinting compared to other printing methods requires the use of low-viscosity bioink that substantially limits types of applicable biomaterials, making difficult to fabricate large-scale 3D structures using this method. In addition, the method ultimately involves extrusion of bioink via the nozzle, which might be clogged during the process. However, as previously mentioned, several advantages compared to other printing methods support the use of the DOD method. Recent studies have actively investigated ways to circumvent its drawbacks. The present review focuses on the DOD inkjet bioprinting technique.

Schöneberg et al. [62] applied the DOD method to simulate multilayer vasculature [Fig. 4B(a)]. A tunica intima with continuous endothelium, a tunica media consisting of elastic SMC layers above tunica intima, and a tunica externa encapsulated with the fibrous and collagenous matrix of fibroblasts were simulated. The maximum thickness and diameter of the fabricated vessel wall were 425 μm and 1 mm, respectively. The tunica intima was printed using gelatin containing HUVECs. After formation of triple-layer blood vessels, gelatin was removed as the sacrificial layer to form hollow vessels. The tunica media was formed using collagen-fibrin-based bioink to reproduce the SMC layer, on which thrombin was added to fibrinogen to induce crosslinking of proteins with fibrin via a calcium-dependent pathway to form the tunica externa [Fig. 4B(a)-i, ii]. The multilayer structure was designed to ensure anatomical resemblance to native blood vessels. By printing different cells (HUVECs, SMCs, and fibroblasts) in each layer, the vascular model was fabricated to exhibit similar physiology to native blood vessels. The addition of an adequate amount of collagen to fibrin reduces the loss of moisture and allows the vascular model to demonstrate excellent mechanical strength. Furthermore, CD31 and VE-cadherin expressions were detected, which implies cell-to-cell interaction and confirms the generation of an endothelial layer. Formation of a network structure was verified based on expression of collagen IV [Fig. 4B(a)-iii] essential for maintaining vascular stability in the core.

Tan et al. [63] applied the DOD method to produce a “mold” to fabricate vascular structures rather than directly printing cell-containing tubular structures. A tissue spheroid hold (mold) is produced, and spheroids are seeded on the interior to fabricate tubular structures followed by removal of the mold. The mold is produced using DOD inkjet printing with 3% (w/v) Alg hydrogel, by which geometrically varying shapes (cube, square frame, and pyramid) can be generated. The mold used to fabricate tubular structures is a ring-shaped biocompatible and bio-inert Alg mold produced via crosslinking of Alg-based microdroplets. In addition, through seeding of spheroids combining human mesenchymal stem cells (hMSCs) and HUVECs at a 1:1 ratio in the mold, interconnected 3D structures are fabricated after culture. Finally, removal of Alg-based mold leads to formation of tubular structures.

While various 2D structures have been fabricated using DOD inkjet bioprinting [64], no study has reported fabrication of complex 3D shapes without adjuncts. As each layer involves droplets, creating free shapes is more challenging than with other printing methods. Nevertheless, more complex shapes can be fabricated when the printing applies a support bath. More details will be provided in the next review. The current review is focused on the method of fabricating tubular structures of higher complexity via deposit of droplets in a support bath.

Xu et al. [65] used DOD inkjet printing in a crosslinker-containing bath to fabricate 3D tubular structures with zigzag shapes [Fig. 4B(b)]. As Alg hydrogel was used as the main bioink material, printing was tried in a bath containing CaCl_2_ solution for immediate crosslinking after printing [Fig. 4B(b)-i]. Droplet conditions and printing parameters are critical during fabrication of 3D structures via DOD printing. At 45 V and a pulse of 3 μs, droplets of moderate size (106 μm) and shape were produced. With a head speed of 200 mm/min, the printing generated droplets with no interval but uniform width [Fig. 4B(b)-ii]. At an overhang angle of 63° for 10-mm structures, printing led to the production of complete overhang tubular structures due to buoyancy of CaCl_2_ solution in the bath with simultaneous crosslinking during printing [Fig. 4B(b)-iii].

Similarly, Christensen et al. [66] printed fibroblast-encapsulated Alg bioink in a CaCl_2_ bath to create a bifurcated vascular tree [Fig. 4B(c)] and more complex structures by combining horizontal and vertical branched structures [Fig. 4B(c)-i, ii]. Due to differences in density between CaCl_2_ solution and Alg hydrogel, printed Alg droplets were supported via gelation [Fig. 4B(c)-iii].

As mentioned at the outset, the bioink used in DOD inkjet bioprinting is limited with respect to viscosity, thus preventing fabrication of complex and precise 3D structures. However, 2 studies [65,66] have demonstrated that complex shapes could be created using a support bath or a sacrificial mold in DOD inkjet bioprinting. Moreover, as these studies could fabricate branched vascular structures, vascular structures with greater geometrical and biological resemblance to native blood vessels could be produced. As such, the DOD inkjet bioprinting technique having high economic efficiency and compatibility with various cells compared to other bioprinting methods has advanced rapidly owing to extensive research. It is one of the most promising techniques in bioengineering.

### Support bath-based bioprinting

**Fig. 5.**
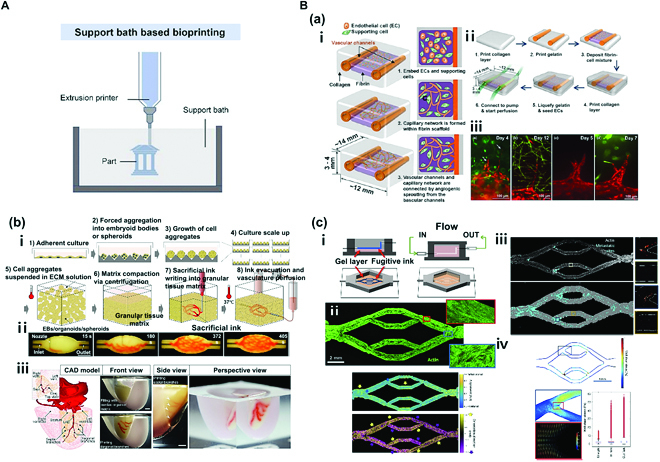
(A) Schematic illustration of support bath-based bioprinting. Reproduced with permission [67]. Copyright 2022, The Authors. (B) Support bath bioprinting: (a) Fabrication of tubular tissue by applying droplet bioprinter to printing. (i) Schematic of growth and maturation process of multiscale vascular system fabricated using 3D bioprinting technology. (ii) Channel construction and fibrin deposition procedure performed using a 3D bioprinter. (iii) Different types of angiogenic sprouts. Reproduced with permission [69]. Copyright 2014, Biomedical Engineering Society. (b) Fabrication of perfusable vascular network using organ building blocks (OBBs), organoids, as a suspension. (i) Step-by-step illustration of the fabrication process. (ii) An image sequence showing embedded 3D printing of a branched, hierarchical vascular network within a compacted EB-based tissue matrix connected to inlet and outlet tubes (scale bar = 10 mm). (iii) A PDMS mold (1:2 scale) was formed using 3D computed tomography data. A left anterior descending artery, together with diagonal and septal branches, was embedded into a septal–anterior wall wedge of the cardiac tissue matrix (scale bar = 5 mm). Reproduced with permission [70]. Copyright 2019, The Authors. (c) Fabrication of perfusable vascular network by applying microextrusion bioprinter to suspension-based printing. (i) Schematic representation of vascular bed printing using sacrificial ink. (ii) Cytoskeletal morphology of microvascular endothelial cells and alignment of cytoskeleton in perfused engineered beds. The preferential alignment follows flow direction. (iii) Full endothelialized vascular bed with identified CTC (circulating tumor cell) sites (cyan). Full acellular vascular beds exhibited a higher CTC burden than endothelialized beds (4% versus 2% of vessel area). (iv) WSS (wall shear stress) and laminar flow during CTC perfusion. WSS profile from vessel sidewalls at center Z slices gathered from simulation data was generated with parameters matching those used for CTC perfusion experiments. Reproduced with permission [71]. Copyright 2020, The Authors.

Both previously described microextrusion and inkjet bioprinting methods lead to the development of 3D structures via simple additive manufacturing of materials on 2D planes. Hence, printing structures with more complex and precise shapes using only the respective bioprinters without adjuncts remains a challenge. Moreover, production of shapes according to viscosity and properties of materials is limited, while structural modification could occur in the mid of the printing process. In addition, materials that can be used in printing to mimic native tissues with respect to mechanical properties are limited. For instance, commonly used materials in bioprinting such as thermoplastic elastomers and silicone rubbers are easily modified by gravity during printing, thereby affecting the printing of large and complex structures. To circumvent this issue, a method using support bath has been developed, in which structures are supported during printing (Fig. 5A) [67]. This method is commonly known as freeform reversible embedding of suspended hydrogels (FRESH)-based bioprinting. It is frequently used in fabricating hollow tubular structures. It allows the use of materials with very low viscosity (e.g., collagen and fibrinogen) [68]. Highly efficient fabrication of complex 3D structures is possible through this method as it involves fixation of the extruded bioink with low mechanical properties by exploiting buoyancy of printed structures or state change of sacrificial materials. Materials typically used in the support bath are those capable of crosslinking, including thermosensitive materials (e.g., collagen, gelatin, and PF 127), pH-sensitive materials, and divalent cations as well as materials whose properties could be altered by ultraviolet (UV) irradiation. The properties of these materials can be modified by external factors such as temperature, pH, UV, and ions to exert a preventive effect on gravity-mediated reduction of shape retention of structures [39]. As these materials are present in a solid or gel state in the bath, a desired shape can be printed using various bioinks inside the bath. Subsequently, the bath can be removed when the printed product no longer requires support. The fabrication could also involve production of a sacrificial mold into which cell encapsulated bioink could be injected for casting. In this review, cases of fabricating a vascular tissue network or complex vascular tubular structures via the use of microextrusion bioprinting and inkjet bioprinting in a support bath have been discussed.

Lee et al. [69] used a collagen layer as the supporting material and deposited gelatin-based droplets on the layer to fabricate perfusable tubular structures [Fig. 5B(a)]. Two gelatin-based vessels were printed on a collagen layer, which were connected indirectly by a capillary bed combining fibrinogen, thrombin, HUVECs, and normal human lung fibroblasts (NHLFs) with another collagen layer covering vessels. After 20 to 30 min of incubation, gelatin in the interior will change to liquid state to produce hollow channels. HUVECs were seeded in these channels, and the medium was perfused for the subsequent culture [Fig. 5B(a)-i, ii]. Sprouts were detected on approximately day 3 of culture. They slowly penetrated the interior of the collagen layer. Moreover, integration of sprouts and a capillary network of the bed were observed, along with angiogenic sprouts of varying shapes [Fig. 5B(a)-iii]. The sacrificial material used in the study was tube-shaped gelatin generated via droplet printing. The collagen layer was used as a support material to fabricate perfusable endothelialized fluidic channels. Particularly, the capillary network between the 2 vessels created a microvasculature connecting the vessels to verify that multiscale vascular networks could be produced.

Skylar-Scott et al. [70] used organ building blocks (OBBs) organoids comprising patient-tailored induced pluripotent stem cells (iPSCs) to develop perfusable vessels [Fig. 5B(b)]. Generated tissues showed high physiological perfection as the team used bioprinting technology and embedded thousands of OBBs with high cell density around perfusable vascular channels. Live OBBs were placed in a mold and compressed via centrifugation to produce a matrix. The gelatin-based sacrificial bioink in the matrix was then patterned using embedded printing to create a branched vascular network. In addition, removal of the sacrificial bioink on the interior after the printing process led to formation of perfusable vascular channels inside [Fig. 5B(b)-i, ii]. Interestingly, when these fabricated vascular channels were cultured for a long period, cell viability was higher in these models with channels than in those without channels. Moreover, when culture involved HUVECs, formation of endothelium was confirmed. Their study showed that a functional and perfusable cardiac structure could be created using iPSC-derived cardiomyocyte OBBs [Fig. 5B(b)-iii]. The resulting small cardiac organoid started beating on day 7 of culture in response to electrical stimulation. Their study is a representative example of not only fabricating an in vitro model that is anatomically similar to native cardiac tissue using bioprinting technology but also imitating physical characteristics of the tissue.

Hynes et al. [71] fabricated an in vitro vascular model using a microextrusion bioprinter via patterning of a sacrificial bioink (PF 127) on gelatin-fibrin hydrogel [Fig. 5B(c)]. After gelation of the hydrogel, PF 127 was removed to create hollow channels. Human cardiac microvascular endothelial cells (hCMECs) were then seeded on these channels to fabricate an in vitro vascular model [Fig. 5B(c)-i]. When the medium was supplied by a bioreactor, endothelial cells completely covered all channels on day 7 of the culture [Fig. 5B(c)-ii]. In addition, perfusion of tumor cells in these in vitro models showed that the probability of adhesion of circulating tumor cells was high at points of branching with large distribution of endothelial cells. The mechanism was also confirmed through computation simulation [Fig. 5B(c)-iii, iv]. It has been hypothesized that hydrodynamic simulation of perfusable in vitro models can be useful for understanding undiscovered mechanisms involved in cancer metastasis and for drug screening. Their team has developed a blood vessel perfusion device that can predict dynamic flow using a sacrificial material. They also fabricated perfusable in vitro vascular models that could be converged with advanced simulation techniques to analyze mechanisms associated with cancer.

### Laser-assisted bioprinting

**Fig. 6.**
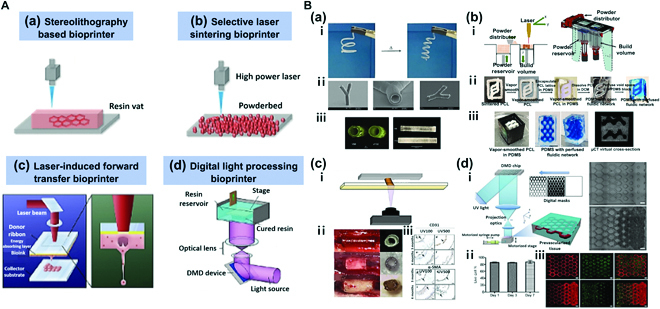
(A) Simplified representation of 4 different categories of laser-assisted bioprinting. (a) Working principle of stereolithography-based bioprinter. (b) Working principle of selective laser sintering bioprinter. Reproduced with permission [72]. Copyright 2016, WILEY-VCH Verlag GmbH & Co. KGaA, Weinheim. (c) Working principle of laser-induced forward transfer bioprinter. Reproduced with permission [74]. Copyright 2019, Elsevier. (d) Working principle of digital light processing bioprinter. Reproduced with permission [75]. Copyright 2020, The Authors. (B) Laser-assisted bioprinting. (a) Fabrication of cylindrical structures using stereolithography and multiphoton polymerization (MPP). (i) Shape memory effect. (ii) Scanning electron micrographs of a branched tubular structure generated by 2-photon polymerization of PTHF-PA 1. (iii) 3D tubes prepared from polytetrahydrofuranether-diacrylate (PTHF-DA) 1 with 0.5% Irgacure-184 and 10% additional crosslinker trimethylolpropane triacrylate. Reproduced with permission [79]. Copyright 2012, The Authors. (b) Fabrication of a perfusable network with a complex structure using OpenSLS bioprinting. (i) Custom open-source selective laser sintering (OpenSLS) hardware. (ii) Process sequence for creating a fluidic network using a sacrificial PCL structure. (iii) Sacrificial templating of a reduced diamond lattice model resulted in formation of a complex, interconnected fluidic network in PDMS, and perfusion with blue dye. Reproduced with permission [80]. Copyright 2016, The Authors. (c) Fabrication of patient-specific implantable vascular grafts using digital light processing (DLP) bioprinting method. (i) Fabrication method using DLP-mediated printing. (ii) Evolution of 3D printed graft over 6 months in vivo. (iii) At 3 and 6 months, an endothelial monolayer was observed despite detachment of the endothelial cell layer during a histological preparation process. Reproduced with permission [82]. Copyright 2015, WILEY-VCH Verlag GmbH & Co. KGaA, Weinheim. (d) Fabrication of vascularized tissues with regular patterns using DLP bioprinting. (i) Schematic diagram of the bioprinting platform (left) with bioprinted acellular construct and cellular construct (right). (ii) Results of cell viability assay of bioprinted tissue constructs encapsulated with HUVECs demonstrating over 85% cell viability. (iii) Fluorescent images demonstrating bioprinting of heterogeneous cell-laden tissue constructs with uniform and gradient channel width (scale bar = 250 μm). Reproduced with permission [84]. Copyright 2017, Elsevier.

Laser-assisted bioprinting is a technique used to print artificial organs and tissues using laser as the energy source. Various types of this technique have been developed, including the following: (a) SLA, a method that can be used to induce localized photopolymerization by laser irradiation on the surface of biocompatible photopolymer resin [Fig. 6A(a)] [72]; (b) selective laser sintering (SLS), a method that can be used to fabricate 3D structures by selective sintering of a photopolymer material in powder form on the bed, followed by heating, binding, and deposition of solidified layers [Fig. 6A(b)] [73]; (c) laser-induced forward transfer (LIFT), a method that applies bioink on a titanium or gold “ribbon” with constant laser pulse on the ribbon layer so that localized bioink on the ribbon layer drops after absorbing energy [Fig. 6A(c)] [74]; and (d) digital light processing (DLP), a method that can print laser reflected through a digital mirror device (DMD) as light corresponding to the single layer is transmitted through photopolymer resin while the plate is gradually lifted following the printing of each monolayer for repeated lamination, resulting in the fabrication of 3D structures [Fig. 6A(d)] [75]. Each method has its own pros and cons. Their shared advantages include absence of a nozzle that avoids nozzle clogging, higher precision than other bioprinting methods, high productivity, and easy fabrication of more complex shapes. Their drawbacks include substantially high cost, a limited range of photopolymer materials, and considerably reduced cell viability compared to other bioprinting methods [76].

Owing to many variations, specific method of laser-assisted bioprinting should be determined based on the characteristics of the target tissue construct. For instance, the LIFT method is advantageous in fabricating soft tissues as it involves laser-based generation of droplets from photosensitive bioink with relatively less potent mechanical properties, followed by droplet deposition [77]. The SLS method is advantageous in fabricating hard tissues as it involves sintering of powder to allow the use of relatively hard materials with rough surfaces [78]. In this section, different methods of laser-assisted bioprinting used to fabricate tubular structures of hard or soft tissues will be discussed.

Meyer et al. [79] applied SLA and multiphoton polymerization (MPP) techniques and used photo-polymerizable α,ω PTHF-DA (polytetrahydrofuranether-diacrylate) resins to fabricate cylindrical structures [Fig. 6B(a)-iii]. In this way, tubular structures with diameter < 2 mm could be fabricated, and a capillary-level supplying system was established. Interestingly, fabricated tubular structures have a feature of shape memory effect. When tubular structures were frozen after fabrication in bent forms at −20 °C and left at ambient temperature, they sprang back to their original shapes [Fig. 6B(a)-i]. Moreover, these tubular structures based on PTHF-DA exhibited ≥87% cell viability with outstanding cell adhesion levels. Additionally, microscale branched tubular structures with an inner diameter of 18 μm and vessel wall thickness of 4 μm were fabricated via 2-photon polymerization with PTHF-PA (polytetrahydrofuranether-polyacrylate) 1 resin [Fig. 6B(a)-ii]. Results showed that SLA and MPP techniques could lead to fabrication of a microscale vascular tubular supplying system as well as vessels with a shape memory effect.

Kinstlinger et al. [80] applied an open-source selective laser sintering (OpenSLS) technique to fabricate a perfusable network of complex shapes. Despite the low accessibility of the LAB (laser-assisted bioprinting) technique due to high cost, an OpenSLS system was developed at a low cost [Fig. 6B(b)], and relatively inexpensive polycaprolactone (PCL) material was used to fabricate 3D overhang structures with sizes <1 mm. The principle of an OpenSLS printer is described as follows. In the powder reservoir, powder is gradually lifted and slowly but steadily pushed to the top part of the build volume by the powder distributor that supplies the powder. Following polymerization of a layer, the piston in the build volume moves back down [Fig. 6B(b)-i]. The process is repeated for deposition to fabricate 3D structures. As structures are formed using powder of very fine particles, the vapor-smoothing technique is applied to polish sintered PCL structures to circumvent the drawback of rough surfaces of printed structures with enhanced elastic modulus and yield stress of the product. Via the OpenSLS system, the utility of bone tissue engineering based on diamond lattice-like porous 3D structures was verified. Respective fabrication of perfusable network was also confirmed [Fig. 6B(b)-ii, iii]. The perfusable network could be made as a sacrificial template for PCL structures fabricated via the SLS process. After forming the sintered PCL of a desired fluidic network shape and surface polishing using the vapor smooth method, the product was inserted to a PDMS mold. Removing PCL using dichloromethane (DCM) leads to a perfusable network without the PCL network in the interior. Hence, this was a representative case of circumventing the drawback of low accessibility due to high cost via development of a low-cost SLS process, which also proved that a sacrificial template could be used to fabricate a complex perfusable network. The network of perfusable complex structures is anticipated as valuable in clinical pathology and drug screening.

Wu and Ringeisen [81] applied the LIFT method to fabricate branch/stem structures with HUVECs and HUVSMCs. The inspiration for designing the vascular network as a leaf vein came from the resemblance between leaf vein and native blood vessel networks. The LIFT process allows seeding droplet into equal intervals. Since there was no thermal damage, shear stress, or rheological damage, cell viability in the live/dead assay approached 100% at 4 days after printing. Furthermore, cell-containing droplets based on 50-μm Matrigel were deposited in 50- to 150-μm intervals to create a vascular network with angiogenesis as with native blood vessels. The complete lumina shape attributed to cell-to-cell connection was confirmed as cells spread out after the first day of culture. In case of HUVECs, cell–cell signaling required a minimum distance of 150 μm between droplets for the formation of a lumen network. Morphological characteristics changed according to the interval and density of deposited cells. This was a representative case of efficient fabrication of interconnected vascular structures via the LIFT method.

Melchiorri et al. [82] applied the DLP bioprinting technique to fabricate implantable vascular structures tailored for patients with congenital heart disease [Fig. 6B(c)]. Vascular grafts were generated via characteristic changes in physical properties upon addition of monoacrylate and a chain transfer agent to PEGDAs [Fig. 6B(c)-i]. Biodegradable poly (propylene fumarate) (PPF)-based material resin was used to form vascular grafts, which were then evaluated by direct in vivo injection. PPF is a biocompatible and biodegradable polyester with carbon–carbon double bonds that can undergo crosslinking in the presence of a photoinitiator. Hence, it is a suitable material for DLP bioprinting. The fabricated implantable vascular grafts demonstrated mechanical strength that could be maintained for a minimum of 6 months. The strength varied according to the level of UV exposure. After PPF vascular grafts were implanted into a mouse venous system, they did not cause stenosis or thrombosis [Fig. 6B(c)-ii]. These printed grafts could be retained in vivo until completion of endothelialization. They assisted growth of the vascular tissue. The study verified the possibility of precision printing of patient-tailored vascular grafts through DLP bioprinting as well as its potential in vivo implantation.

Similarly, Baudis et al. [83] fabricated implantable grafts for narrow blood vessels (coronary bypass) through DLP bioprinting. To form grafts, an elastomeric degradable biomaterial was developed, whereby urethane diacrylate, hydroxyethyl acylate reactive diluents (HEA), and dithiol were combined. The material was prepared by mixing HEA and polyurethane with excellent elasticity and biocompatibility. It displayed an adequate level of mechanical strength. Addition of dithiol increased the content of urethane groups. Combination of the 3 components allowed production of a tailored photopolymer material with degradability and elastomeric properties that resemble the native vascular structure and has an outstanding suture tear resistance. The team applied the DLP technique to CAD (computer-aided design)-CAM (computer-aided manufacturing) to fabricate precise vascular structures and observed that vascular grafts with mechanical properties similar to those of native blood vessels could be produced.

Zhu et al. [84] developed vascularized tissues with regular patterns through DLP bioprinting [Fig. 6B(d)]. Using microscale continuous optical bioprinting (μCOB), a DLP-based printing method, vascular tissues were formed in a remarkably rapid and efficient manner. A digital micromirror array device (DMD) used in printing consisted of approximately 2 million micromirror arrays, whereby the shape of the light projected to the monomer solution was controlled. For removal of the prepolymer solution, a motorized syringe pump system was used [Fig. 6B(d)-i]. The process allowed fabrication of precise and complex structures without using sacrificial materials. To mimic native blood vessels, various cells (HUVECs, C3H/10T1/2) were encapsulated in glycidyl methacrylate-hyaluronic acid-based bioink. The printing generated tubular structures with diameters ranging from 5 to 50 μm. The extremely rapid printing time reduced the time of cellular UV exposure, thereby allowing substantially high cell viability (>85%) [Fig. 6B(d)-ii]. When vascular structures were cultured for 1 week, formation of an endothelial network and combination between α-SMA and HUVECs with lining along vessel walls was observed. Moreover, in vivo implantation of these structures led to the formation of a progressive endothelial network [Fig. 6B(d)-iii]. Their team proved that the μCOB printing, a DLP-based method, could ensure a substantially rapid formation of vascular tissues of complex structures with outstanding resolution, flexibility, and expandability. This case showed that low cell viability associated with the LAB technique, which limited the technical scope, could be circumvented by a high printing speed.

The LAB technique exhibits high resolution and accuracy. It has the advantage of fabricating complex structures compared with other printing methods. Moreover, different methods (DLP, SLS, etc.) can be used to fabricate 3D structures with high mechanical strength that resemble those of the actual human body. Despite drawbacks of high cost and low cell viability due to UV-based polymerization, short time of printing, as demonstrated by the aforementioned cases, has various ways to overcome such disadvantages. The LAB technique is one of the promising methods that may prove to be the most efficient and precise printing method with continuous advancements.

## Conclusion and Future Outlook

BMs can recapitulate the structure and physiology of native human organs and their development and diseases, which can help overcome limitations of 2D in vitro and animal models. BMs are important tools for understanding biological systems and developing new technologies in fields such as medicine and biomanufacturing. However, they have limitations and constraints such as simplified representations. BMs often involve simplifications and abstractions of complex biological systems. These simplifications can limit the accuracy and applicability of the model, particularly if important features or interactions are overlooked. Multiscale modeling involves integrating models of different scales, from molecular to cellular to tissue level, to capture the complexity of biological systems. This can help address the issue of simplified representations and improve the accuracy and applicability of the model. Furthermore, BMs that involve experimentation using living organisms or human subjects raise ethical considerations that must be carefully considered and addressed. In vitro experimentation involves studying biological systems outside a living organism, while in silico experimentation involves computer simulations. In vitro experimentation can help address the issue of ethical considerations by reducing the need for experimentation using living organisms or human subjects. Overall, by incorporating these alternatives and improvement methods, BMs can become more accurate, reliable, and effective tools than 2D in vitro and animal models for understanding biological systems and developing new technologies.

One method of biofabrication for creating BMs is the 3D bioprinting technique, which involves layer-by-layer stacking of a computer-designed form using cells containing bioink. This technique can be used to rapidly print highly elaborate forms with outstanding repeatability and reproducibility. Although this technique is relatively new, endless possibilities and potential for advancement of 3D bioprinting have led to the development of many bioprinting techniques. Each printing technique has its advantages and disadvantages. These techniques allow printing of sophisticated and complex tubular structures of blood vessels compared to other conventional techniques. Moreover, 3D bioprinting holds the advantage of fabricating microscale, ultrafine tubular structures, opening doors to novel applications in tissue engineering and regenerative medicine.

However, there are also a number of challenges to overcome in the field of 3D bioprinting. The availability of biocompatible natural polymers to make bioinks is limited, and some materials are not cheap. In addition, proper curing mechanisms must be utilized to ensure that the bioprinted structure retains its 3D shape, a process that can adversely affect cell survival. Therefore, it is necessary to develop bioink that is inexpensive, is easy to handle, and can actively promote cell survival, adhesion, and proliferation that can mimic and reproduce the human microenvironment. In addition to bioink, there are also technical limitations to bioprinting itself. There are various sizes of human tissues ranging from nanometers to micrometers. To reproduce them in printing, various variables such as printer's head, nozzle, and external forces can be controlled to a certain extent. However, it is not possible to reproduce them perfectly. There are also various technical problems with bioprinters, such as cell damage caused by shear stress generated by the nozzle. Furthermore, although bioprinting has been used to fabricate perfusable vessels with some success through capillary sprouting and angiogenesis, its effects are still unclear in microfluidic studies. Angiogenesis and vasculogenesis that integrate a microfluidic system with bioprinting as a microchannel could be a successful strategy to enhance capillary sprouting. This requires optimized bioink with a porous structure so that the cells can expand easily with an adequate oxygen supply. The use of angiogenic growth factors for sprouting and angiogenesis can also be considered. Further research should be conducted to elucidate the underlying mechanisms and optimize bioink composition and microfluidic design to achieve better control and functionality in the development of vascularized tissues. If these limitations are overcome in the future, 3D bioprinting is considered to be an advanced technology that can be widely used in the fields of healthcare, regenerative medicine, drug development, and tissue engineering.

Tissue engineering is a field of research that mimics various human tissues/organs, and many parts of the body are composed of tubular tissues, which have led to the development of several methods to create them in vitro. Tubular tissue structures are found throughout the human body, including vascular (artery, vein, and capillary), respiratory (trachea and bronchus), digestive (esophagus and stomach), and urinary (ureter and urethra) systems. The vascular system plays a critical role in delivering oxygen- and nutrient-containing blood throughout the body and absorbing and excreting carbon dioxide and waste produced via cellular metabolic activities. Given the vital role of the vascular system in general activities of the body, vascular diseases can cause severe physiological dysfunctions.

An in vitro simulation of blood vessels allows drug screening for diseases related to blood vessels (such as coronary artery disease, peripheral artery disease, vasculitis, atherosclerosis, and thrombosis). This simulation also permits a causal analysis based on real-time monitoring of incurable and intractable diseases such as cancer, cerebrovascular disease, and CVD, which are directly or indirectly associated with the vascular system. Various tissue engineering techniques have been investigated for the construction of in vitro vascular models, including sheet rolling, tubular molding, dip coating, and sheet assembly. These techniques are advantageous because they enable sound and simple fabrication of tubular structures. However, there are challenges associated with the fabrication of tubular structures with very small diameters, such as capillaries, as well as the production of realistic in vitro models of tissues with branched forms or structures combined with blood vessels or other tissues. Therefore, advancements in scaffold fabrication techniques, such as electrospinning or 3D scaffolding approaches, and fine-tuning printing parameters based on the specific requirements of tubular structures and complex tissue models can contribute to the creation of more intricate and tailored structures.

Bioprinting can be used to fabricate blood vessels with complex, microscale structures in vitro for the construction of in vitro models featuring multiple connected tissues. Additionally, fabrication of vascularized tissues that closely resemble anatomical tissues will allow a detailed in vitro examination of diseases related to blood vessels and further large-scale fabrication of diverse, large-caliber tissues. Recent rapid advancement of techniques to fabricate in vitro models is expected to overcome current limitations, paving the way for more accurate drug evaluation and efficacy analyses of blood vessels and blood flow dynamics in the body. Furthermore, expected results will benefit organ transplantation, the demand for which far exceeds the number of donors.
